# Vertebral body fractures of unknown origin in cancer patients receiving MDCT: reporting by radiologists and awareness by clinicians

**DOI:** 10.1186/s40064-016-2097-5

**Published:** 2016-04-14

**Authors:** Melanie Wild, Peter Dankerl, Matthias Hammon, Michael Uder, Rolf Janka

**Affiliations:** Department of Radiology, University Hospital Erlangen, Maximiliansplatz 1, 91054 Erlangen, Germany; Department of Orthopedic Surgery, Klinikum Forchheim, Krankenhausstraße 10, 91301 Forchheim, Germany

**Keywords:** Osteoporosis, Vertebral body fracture, Pathologic fractures, Radiological reports, Clinical management, Cancer patients

## Abstract

**Background:**

To evaluate prevalence, radiological reporting and clinical management of pathologic vertebral body fractures (VBFs) of unknown origin in cancer patients receiving computed tomography (CT) examinations.

**Methods:**

We investigated all CT examinations (over 1 year) of male and female patients with an underlying malignancy and an increased risk of osteoporosis (age 55–79 years) for the presence of VBFs. We evaluated midline sagittal CT-reformations of the spine for prevalence, fracture type, severity and location, the accuracy and style of radiological reporting, subsequent clinical management and documentation in hospital discharge letters.

**Results:**

848 patients were investigated. We found 143 VBFs in 94 (11 %) patients. 6, 49, and 45 % were grade 1, grade 2, and grade 3 fractures, respectively, while 20, 66, and 14 % were wedge, biconcave and crush fractures, respectively. 32 (34 %) radiological reports correctly classified VBFs as fractures, 25 (27 %) reports recognized VBFs, but did not type them, and VBFs were not described in 37 (39 %) reports. In 3 (3 %) patients further clinical work-up of VBFs was performed, while only 8 (9 %) hospital discharge letters contained the information of the presence of pathologic VBFs of unknown origin.

**Conclusions:**

VBFs of unknown origin appear frequently in cancer patients, however, clinical management and documentation was found in only few cases. Moreover, especially in cancer patients consistent radiological reporting of VBFs seems important, as aetiology of VBFs could be from osteoporosis, disease progression or oncological therapy, however, reporting is still performed inconsistently.

## Background

**Fig. 1:**
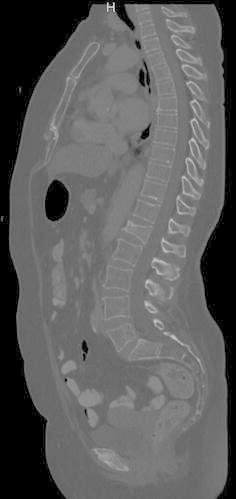
Midline sagittal reformatted CT image of a 71-year-old woman with a follow-up examination for malignant melanoma class IIIB. Vertebrae C7 to L5 were completely pictured in the computed tomography examination and were evaluated in this study. A typically appearing pathologic VBF of the first lumbar vertebrae grade 2, type wedge can be appreciated. This was reported in the impressions section of the initial radiological report; however, it was not documented in the patient’s discharge letter and, the underlying cause, be it osteoporosis or metastasis has not been determined

Pathologic fractures are fractures of “diseased and weakened bone” and occur without adequate trauma (Curtis et al. 2009). In the general population most cases of pathologic vertebral body fractures (VBFs) are caused by osteoporosis (Jung et al. 2003) and typically present as compression fractures. However, especially in cancer patients presenting with VBFs of unknown origin, osseous metastasis has to be considered, even if the CT-morphological appearance is that of a typical osteoporotic compression fracture (Fig. 1).

Vertebral body fractures are the most frequent type of osteoporotic fractures, and they occur significantly earlier compared with wrist and hip fractures (Sambrook and Cooper 2006). Typically, hip fractures are the most serious manifestation of osteoporotic fractures, causing persisting disabilities, excess mortality, and great initial hospitalization and operation costs (Melton and Cooper 2009). However, after sustaining an osteoporotic fracture, patients are at a 50–100 % greater risk of suffering another osteoporotic fracture (Klotzbuecher et al. 2000). Therefore, VBFs are viewed as osteoporotic indication fractures. This offers the chance of preventing further and more serious hip fractures by treating the underlying osteoporosis in patients with newly developed VBF.

Over a decade ago the International Osteoporosis Foundation developed a Vertebral Fracture Initiative to educate radiologists and to raise awareness for the relevance of detecting and reporting VBF (Schwartz and Steinberg 2005). However, previous investigations concerning radiological reporting of VBFs (Carberry et al. 2013; Bartalena et al. 2009; Müller et al. 2008) excluded patients with known malignancies from their investigations. However, a recent, large epidemiological study found that excluding patients with an underlying malignancy from research on pathologic fractures underestimated the incidence of VBFs from osteoporosis (Curtis et al. 2009). Furthermore, the current European Society of Medical oncology guideline advises pharmacotherapy for cancer patients with osteoporosis (Coleman et al. 2014) Therefore, pathologic fractures in cancer patients have to be evaluated and worked-up either for the presence of osteoporosis or for osseous metastasis. Due to the retrospective nature of our investigation we cannot determine the cause of the pathologic VBF, be it osteoporosis or metastasis—however this was not our investigational focus. Fur pure purpose of detection and reporting, the aetiology of VBFs is of lesser significance for the radiologist. However, when considering patient care and wellbeing from a radiological and especially clinical standpoint, aetiology of VBFs is of great importance. In fact, the unknown origin of VBF even more so should lead the clinician to further investigate and properly document the incidental fracture, as it can be induced by osteoporosis, cancer or oncological therapy (e.g., radiation or drug induced).

The aim of our research was to retrospectively investigate the prevalence and radiologists’ performance to detect and report VBFs of unknown origin in routine CT examinations in cancer patients, and investigate subsequent clinical management and documentation of VBFs.

## Methods

Institutional review board approval was obtained for the retrospective analysis, and the need for informed consent was waived. All procedures were in accordance with the Helsinki Declaration.

### Study population

A query in the radiological information system (RIS) with the following constraints was performed: 12-month period; patient age 55–79 years; 64-row CT examination containing the abdomen and pelvis (abdomino-pelvic, thoraco-abdomino-pelvic, or cervico-thoraco-abdomino-pelvic CT-examinations). Patients with previously known osseous spinal metastasis were excluded. Furthermore, patients with a recent history of trauma—in order to exclude patients with traumatic VBFs, were excluded as well. Images and radiological reports from 1229 CT examinations were retrieved. From these, 381 patients did not demonstrate an underlying malignancy, and were excluded from further investigation. All remaining 848 CT examinations (296 women and 552 men; mean age 65.2 years ± 6.1) were included in the evaluation.

### Image acquisition

CT examinations were indicated by clinical needs in all cases. All patients were examined with a 64-row CT scanner (Somatom 64, Siemens AG, Erlangen, Germany; collimation 0.6 mm). From the raw data, sagittal images with a slice thickness of 3 mm and an increment of 3 mm were reconstructed utilizing a sharp (bone) kernel (70f). These images were present in the initial reading and were reevaluated.

### Image analysis

Images were investigated utilizing a standard patient archiving and communicating system (PACS) workstation with dual high-resolution/high-brightness monitors. After specific training, a resident (XX) who was blinded to the initial reports analyzed the reconstructed sagittal images of all 848 patients in a bone window setting (level 1000; width 3000) for the presence of VBF utilizing the Semi-Quantitative (SQ) method (Genant et al. 1993). To exclude rather subjective false positives, especially grade 1 VBFs, the resident further performed 6-point vertebral morphometry (Kleerekoper et al. 1984) on all suspected vertebrae, and only verified fractured vertebrae were further investigated.

As a reference standard, experienced readers (PD and MH) consensually reviewed all suspected vertebral fractures by applying the Algorithm-Based Qualitative (ABQ) (Jiang et al. 2004; Ferrar et al. 2008) assessment. If CT-morphological criteria were suspicious of obvious malignant fractures (cortical disruption, lytic/sclerotic lesions or soft tissue component; present in 12 patients with VBF) these patients were excluded. If CT-morphological criteria indicated vertebral deformities (e.g., from Scheuermann’s disease, limbus vertebra, or cupid’s bow contour in corda dorsalis remnant; present in 21 patients with initially incorrectly classified VBF) these patients as well were excluded from the study population.

Fracture severity was categorized into grade 1 (20–25 % height reduction), grade 2 (25–40 % height reduction), and grade 3 (>40 % height reduction), and fractures were classified as wedge, biconcave, or crush fractures.

### Patients’ medical record review

After image analysis, the original radiological reports of all patients with VBFs were examined to determine whether the fractures were reported prospectively. The radiological reports were evaluated according to the guidelines of the IOF and ESSR (Genant and Bouxsein 2015). They claim to explicitly name every VBF, independent of grade or type, as a fracture. The findings and conclusions sections of each report were examined for any description or assessment of VBFs, and the wording was documented. Moreover, patients’ hospital discharge letters were evaluated for documentation and or further workup of VBFs of unknown origin.

### Statistical analysis

Statistical analysis was performed using dedicated software (SPSS Statistics v20, IBM Corp., Armonk, NY, USA). Descriptive statistics of patient specifics, fracture analysis, accuracy and style of reporting in radiological reports, and documentation in hospital discharge letters were performed.

## Results

**Fig. 2:**
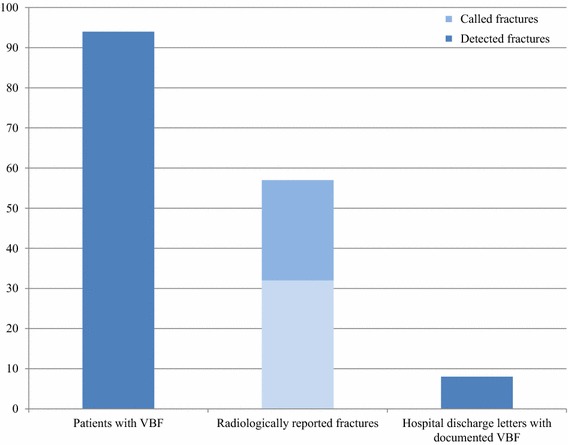
Histogram displaying patients with vertebral body fractures/VBFs (94), patients being reported as having “fracture” (*lighter blue*) or VBFs being described semantically (*darker blue*), and number of patients with documented VBFs in their hospital discharge letters

289 cervico-thoraco-abdomino-pelvic, 314 thoraco-abdomino-pelvic, and 245 abdomino-pelvic CT examinations of 848 patients were included and further evaluated. In 152 (17.9 %) cases evaluated CTs detected primary tumors, and in 696 (82.1 %) CT was indicated for response evaluation and follow-up of known tumors and metastases. Collectively, 15.298 individual vertebrae were evaluated for the presence of VBFs. Detailed information concerning the fracture locations is shown in Fig. 2.

Overall, 143 (0.9 %) VBF were detected in 94 (11.1 %) patients (mean age 66.4 ± 6.2 years) in 33 (35.1 %) women and 61 (64.9 %) men. Most fractures, 52 out of 143 (36.4 %) were found at the thoracolumbar junction (T11-L2), the pathognomonic location for VBFs. 2 (1.4 %) fractures were detected in the cervical spine (C1–C7), while 18 out of 143 (12.6 %) were found in the upper thoracic-spine (T1–T6). The most often fractured vertebral bodies were L1 (21 out of 143 (14.7 %) and L5 (19 out of 143 (13.3 %). 8 out of 143 (5.6 %) were grade 1 fractures, 71 out of 143 (49.6 %) were grade 2, and 64 out of 143 (44.8 %) were grade 3 fractures. 29 out of 143 (20.3 %) of the fractured vertebrae were wedge fractures, 94 out of 143 (65.7 %) were biconcave fractures, and 20 out of 143 (14 %) were crush fractures.

In 32 (34 %) out of 94 radiological reports, VBFs were classified as fractures, either in the description or the conclusion section. In an additional 25 (27 %) reports, VBFs were recognized and described in various texts in the description section; however they were not named *fracture*. Instead, characterizations such as “reduced vertebral height”, “baseplate impression”, “degenerative height reduction”, “degenerative baseplate changes”, or “sintering”, were found in the description section of the radiological reports and were not mentioned in the “Conclusions” section. 37 (39 %) VBFs were not described at all in the initial radiological report. Collectively, 14 different radiological attending physicians and residents prepared the initial evaluated radiological reports.

Of the 94 patients featuring VBFs of unknown origin, only 3 (3 %) patients received further clinical management of VBFs (one received an MR, and two patients received t-score measurements), while hospital discharge letters in 8 (9 %) patients included the information of VBFs. Indeed, in all of these 8 cases, VBF were called *fractures* in the conclusion section of the radiological reports.

## Discussion

As presented, 94 out of 848 (11 %) patients with cancer undergoing routine CT examination demonstrated one or more VBFs of unknown origin. In a study on abdomino-pelvic CT examinations (cohort of 2041 patients age 19-94) Carberry et al. (Carberry et al. 2013) found a prevalence of 4.8 % for vertebral body compression fractures. Two previous studies with smaller patient cohorts [323 patients age 20–88 Bartalena et al. (2009) and 112 women age 55–87 and Müller et al. (2008)] that investigated VBFs in thoraco-abdomino-pelvic CT examinations found prevalences of 9.5 and 24.1 % respectively. However, all these studies excluded patients with known malignancies from their investigations. Compared to these data pathologic VBFs seem to appear at least as frequently in cancer patients as in patients without underlying malignancy. However, consistent radiological reporting and clinical awareness seem to be a problem. We deliberately excluded 12 patients with fractures which were obviously caused by osseous metastasis (several lytic/sclerotic vertebral lesions; soft tissue component of the fractured vertebra), as we only wanted to examine typically appearing vertebral body compression fractures (Fig. 1). These pathologic VBFs of unknown origin are difficult for radiologists and clinicians for various reasons. For one, many patients have osteoporosis and feature VBFs which can lead radiologists to rate these fractures as an insignificant finding, especially in older patients with known osteoporosis. For another reason, to the clinician a VBF might most likely be caused by osteoporosis and therefore might seem less relevant than the development of the underlying malignancy—the reason the CT was ordered in the first place. Nevertheless the aetiology of typically appearing pathologic VBFs of unknown origin especially in cancer patients is uncertain, which makes it even more urgent to either diagnose or exclude osteoporosis as some of the typically appearing VBFs might be caused by metastasis or oncological therapy (radiotherapy or drug related). Interestingly, only 3 patients received further workup of VBFs of unknown origin, while 8 patients had this diagnosis documented in their discharge letters.

We demonstrated that in 32 patients, the VBFs were explicitly named fractures in the radiological report and another 25 VBFs were described; however, they were not named fractures. In 8 of these 57 patients for whom VBFs were detected in the initial reading, the medical record contained this information. This leaves two interpretations: either the clinicians did not take notice of the fractures, or the information did not seem important enough to initiate further diagnostic workup. However, this increases the importance of naming every VBF a fracture in the radiological conclusion section, which most likely is the only section reviewed by the treating physician when preparing the patients’ discharge letter.

It is scientific consensus that VBFs are underdiagnosed in radiographic examinations, with reported detection rates ranging from 55 % (Kim et al. 2004), all the way to 9 % (Oschatz et al. 2009) while Gruber et al. (2013) found an intermediate detection rate of 28 %. Furthermore, reporting of VBFs in CT examinations has been done in even fewer cases, especially those without sagittal reformations, displaying detection rates of 16, 15 and 13 % respectively (Carberry et al. 2013; Bartalena et al. 2009; Williams et al. 2009). Furthermore, only 35 % of radiographically reported VBFs were shown to be documented in hospital discharge letters (Gehlbach et al. 2000). Now, one decade after the Vertebral Fracture Initiative (Schwartz and Steinberg 2005), we found improved detection rates (61 %), most likely as a result of the routine reading of sagittal reformations. Nevertheless, clinical management rates of pathologic VBFs of unknown origin detected in CT examinations of cancer patients were an inadequate 3 %. Our findings are supplemental to those of Hernlund et al. (2013), and they show that not only the majority of patients with osteoporosis, but patients with osteoporosis and cancer, are untreated for osteoporosis. Furthermore, unreported and unmanaged incidental VBFs in cancer patients may as well represent oncological disease progression, making an investigation and management even more important. Bartalena et al. (2010) stated that radiologists did not learn their lessons in detecting and reporting vertebral fractures. As presented, this statement is only true in about 50 % of cases, which, however, is far away from radiological reporting rates for other diseases and findings.

One problem might be the fact that radiologists cannot diagnose osteoporosis on routine CT examinations, only osteopenia. Instead, to date for the correct diagnose of osteoporosis dedicated quantitative CT or X-ray is required (Link 2012). However, software-driven solutions (e.g., similar to Fidler et al. 2015) for the automatic evaluation of bone mineral density can aid the radiologist in diagnosing osteoporosis on routine CT examinations. Hence, if osteoporosis can be diagnosed routinely on non-dedicated CT examinations—much like an add-on, and VBFs are detected, perhaps radiologists and clinicians will become more aware of the need to report and manage VBFs of unknown origin correctly.

There are some limitations to our research. Firstly, our standard of reference comprises of CT-morphological image criteria and a consensus read, in order to exclude obvious malignant VBFs and vertebral deformities from our study population. Furthermore, as we do not have a clinical workup of patients with VBFs, we cannot account for the number of fractures caused by osteoporosis, malignancy, or oncological therapy related, however, this was not the focus of our work. Furthermore, as L5 is not the typical location of an osteoporotic fracture and in our work 13 % of fractures were located here, these as well might be secondary due to medical treatment. Indeed, cancer patients often receive high cortisone doses or have had a neoadjuvant radiation in rectal cancer, possibly explaining the high prevalence of L5 fractures. On the other hand, the unclear nature of the pathologic VBF should entice the clinician even more so to work up and this finding. Indeed, a missed and unmanaged metastasis causing VBF might have an even worse impact on patient morbidity and mortality than a missed and unmanaged osteoporotic VBF.

## Conclusions

VBFs of unknown origin appear frequently in cancer patients, however, clinical management and documentation was found in only few cases. Moreover, especially in cancer patients consistent radiological reporting of VBFs seems important, as aetiology of VBFs could be from osteoporosis, disease progression or oncological therapy, however, reporting is still performed inconsistently.

## Abbreviations

CT: computed tomography
VBF/VBFs: vertebral body fracture(s)
RIS: radiological information system
PACS: patient archiving and communicating system
SQ: semi-quantitative
ABQ: algorithm-based qualitative
IOF: international osteoporosis foundation
ESSR: European society of musculoskeletal radiology
